# Molecular-Phylogenetic Characterization of the Microbiota in Ulcerated and Non-Ulcerated Regions in the Patients with Crohn's Disease

**DOI:** 10.1371/journal.pone.0034939

**Published:** 2012-04-18

**Authors:** Qiurong Li, Chenyang Wang, Chun Tang, Ning Li, Jieshou Li

**Affiliations:** 1 Research Institute of General Surgery, Jinling Hospital, Nanjing, Jiangsu, People's Republic of China; 2 Department of Surgery, School of Medicine, Nanjing University, Nanjing, Jiangsu, People's Republic of China; Charité, Campus Benjamin Franklin, Germany

## Abstract

**Background:**

The dysbiosis of intestinal microbiota has been established in Crohn's disease (CD), but the molecular characterization of this dysbiosis in Chinese subjects with CD remains unclear. This study aims to investigate the predominant bacterial composition of the faecal and mucosal-associated microbiota in Chinese CD patients using culture-independent techniques.

**Methods/Principal Findings:**

Eighteen patients with CD and 9 healthy controls were included in this study. The faeces and the intestinal mucosal tissues from the ulcerated and nonulcerated sites were subjected to bacterial community fingerprinting using denaturing gradient gel electrophoresis (DGGE). The predominant bacterial composition in the faeces and mucosa was determined with DNA sequencing and BLAST. We showed that the bacterial diversity in the faeces of CD patients was reduced compared with that in healthy controls (*p*<0.01). The faecal bacterial dysbiosis of the patients was characterized by an elevated abundance of γ-Proteobacteria (especially *Escherichia coli* and *Shigella flexneri*) and a reduced proportion of Bacteroidetes and Firmicutes. Five bacterial species defined the microbiota imbalance of the ulcerated mucosa in CD, including an increase in *Escherichia coli*, a decrease in *Faecalibacterium prausnitzii*, *Lactobacillus coleohominis*, *Bacteroides sp* and *Streptococcus gallolyticus* in the bacterial community as compared with the nonulcerated (*p*<0.01).

**Conclusions/Significance:**

This is the first description of intestinal microbiota dysbiosis in Chinese CD patients. These results allow a better understanding of the faecal and mucosal microbiota in CD, showing a predominance of some opportunistic pathogenic bacteria and a decrease in beneficial bacterial species. The findings may provide novel insights into the pathogenesis of CD in Chinese population.

## Introduction

Crohn's disease (CD) is a chronic, recurrent inflammatory disorder of the gastrointestinal tract frequent in Western countries [1]. In recent years, CD incidence has been increasing in China [2], [3]. However, its aetiology is still unclear. CD is defined as a complex trait that results from the interaction between the host's genetic background and the resident commensal microbiota [4]–[7]. Recent studies suggest that changes in the gut microbiota play a part in the pathogenesis of CD [8]–[10]. The role of the commensal microbiota in the onset and perpetuation of the intestinal inflammation in CD is well established [11], [12]. A global decrease in the biodiversity and a markedly reduced diversity of Firmicutes, and in particular of the *Clostridium leptum* phylogenetic group are described as the specificities of the fecal microbiota in CD [13]–[15]. The composition of the mucosal and faecal microbiota has been demonstrated to be significantly different [16], [17]. The mucosal microbiota is of particular interest because the mucosal surface is the site of microbial recognition by the host [18]. CD is a disorder of mucosal inflammation and the mucosa-associated microbiota seems of peculiar relevance to the disease process [18], [19]. Abnormal composition of mucosa-associated microbiota in patients with CD has been found [18]–[20]. CD has increased amounts of bacteria attached to their gastrointestinal epithelial surfaces compared with healthy individuals and a proportional abundance of mucosa-associated *Escherichia coli* and *Pseudomonas* species [21]–[24]. *Faecalibacterium prausnitzii* was particularly depleted in ileocolonic mucosa-associated microbiota in CD [24], [25].

The human intestinal microbiota is a complex ecosystem containing hundreds of microbial species with a density exceeding 10^12^ bacteria per gram and it contributes to the host's nutrition, intestinal epithelial renewal and development of immune response [26], [27]. It is well established that the pathogenesis of CD is linked to microbial colonization, host genetics, environmental factors and immune dysfunction [4], [28]–[30]. Because the composition of the intestinal microbiota is likely to be influenced by genetic and environmental factors as well as dietary habits [16], [31], [32], the abnormal response of intestinal microbiota in CD might be driven by the genetic background and dietary custom in the host. Although supported by little evidence, the composition of intestinal microbiota in Chinese CD population may differ from that in Western countries. The changes of intestinal microbiota in Western population with CD have been broadly studied. However, the characterization of alterations in the composition and diversity of the intestinal microbiota in CD of Chinese population remains unknown.

The aim of the current study was to carry out a molecular analysis on the composition and diversity of the intestinal microbiota in Chinese patients with CD using denaturing gradient gel electrophoresis (DGGE) and DNA sequencing. This study was to compare the microbiological profiles of the faeces from CD patients and healthy controls and to identify the bacterial species characteristic of CD patients. We also determined the predominant bacterial composition of the mucosa-associated microbiota in the ulcerated ileum of CD patients. Our study was the first to define the changes of the intestinal microbiota in Chinese CD patients, which might provide novel knowledge on the role of intestinal bacterial dysbiosis in CD pathogenesis of Chinese population.

## Methods

### Patients

Eighteen patients (male/female: 10/8) with ileal CD and 9 healthy subjects (male/female: 5/4) were enrolled in this study. The median (range) ages were 35.2 (18–58) years for CD group and 33.7 (20–62) years for the controls. All participants were native residents in the mainland of China. The diagnosis of CD was made in accordance with established clinical, endoscopic, radiological, and histological criteria [33]. Disease activity was assessed by the Crohn's disease activity index (CDAI) [34] and all patients were in an active phase of disease (CDAI>150). Subjects taking antibiotics or probiotics within 4 weeks before sampling were excluded from the analyses.

The Ethics Committee for Human Medical Research of Jinling Hospital approved this study, and all participants gave written informed consent. Faeces were collected with sterile container from CD patients and healthy volunteers. The ileal mucosal specimens including the ulcerated and nonulcerated areas were surgically obtained from the patients. The tissue specimens were washed three times with normal saline and then stored at −80°C for DNA extraction.

### DNA Extraction and Polymerase Chain Reaction (PCR)

DNA extraction of faecal samples was performed using a QIAamp DNA Stool Mini Kit (QIAGEN, Valencia, CA, USA) according to the manufacturer's instruction. Mucosa-associated bacterial DNA was extracted from ileal tissue sections by QIAamp DNA Mini Kit. The extracted DNA was checked by agarose gel electrophoresis (1% w/v) and were quantified spectrophotometrically. The hypervariable V3 region of 16S ribosomal RNA gene was amplified using PCR with universal bacterial primer sets (F357+GC clamp and R518) [35].

### DGGE

DGGE was performed using a DCode universal mutation system (Bio-Rad, Hercules, CA, USA). The sequence specific separation of PCR amplicons was conducted on 8% (w/v) polyacrylamide (acrylamide∶bisacrylamide, 37.5∶1) gels containing a urea-formamide gradient from 35 to 55% as previously described [36]. The electrophoresis was done at 120 V for 7.5 h at 60°C. The bands were stained with SYBR Green I (Invitrogen, Carlsbad, CA, USA) and imaged by UV transillumination with the ChemiDOC™ XRS instrument (Bio-Rad).

### Comparative Analyses of DGGE Profiles

A scanned image of an electrophoretic gel was analyzed using QuantityOne software (Version 4.2, Bio-Rad). The similarities between DGGE profiles were assessed by the Dice coefficient and the unweighted pair group method with the arithmetic average (UPGMA) clustering algorithm, and the corresponding dendrograms were constructed. The relative abundance of each band was quantified by the staining intensity and was expressed as a proportion (%) of the sum of all fragments in the same lane of the gel [37]. The general diversity of bacterial communities was calculated according to Shannon and Wiener, as described previously [38], [39]. Principal component analysis (PCA) plots were generated based on the number of DGGE bands using the multivariate statistics software Canoco (version 4.5, Microcomputer Power, Ithaca, NY, USA).

### Sequence Alignment and Phylogenetic Analysis

To identify the bacterial species correlated with the bands, selected products from the stained gels were excised with sterile scalpels. Gel slices were then incubated in 20 µl of elution buffer overnight at 4°C. Four µl of the resulting DNA solution was used as the template for re-amplification and PCR product was checked by DGGE as described previously [36]. The re-amplified fragments were purified and subjected to automated sequence analysis on an ABI PRISM 3730 sequencing system (Applied Biosystems, Foster City, CA, USA). The retrieved sequences were compared with the Genbank database using BLAST algorithm. Phylogenetic trees were generated via a neighbor-joining algorithm from evolutionary distances through the MEGA software (version 4.0).

### Statistical Analysis

Data are presented as means ± standard deviation (SD). Statistical analysis was performed by Student's *t* test or one-way analysis of variance (ANOVA) followed by the Holme-Sidak test using the SPSS software (version 12.0). A p value of less than 0.05 was considered significant.

## Results

### Shifts in Faecal Bacterial Microbiota

**Figure 1:**
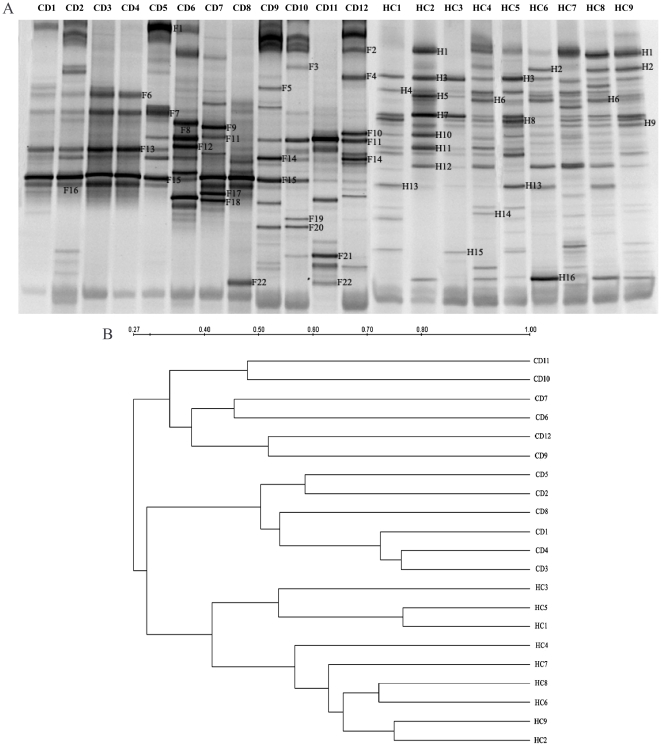
The dysbiosis of faecal microbiota in the patients with Crohn's disease (CD). (A) Representative DGGE profiles of the faecal samples from CD patients and healthy controls. CD represents Crohn's disease patients and HC is healthy controls. (B) Dendrogram illustrating the similarity correlation of fingerprints between the patients and healthy subjects by means of the clustering algorithm of UPGMA. Scale bar describes DGGE similarity between profiles.

**Figure 2:**
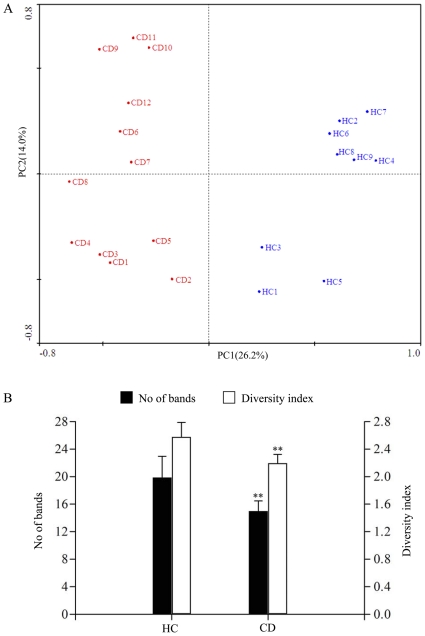
The plots of principal component analysis of microbiota composition and biodiversity from faecal samples. (A) Principal component analysis of the DGGE data for the fecal bacterial community compositions of the CD patients and controls. Each circle is representative of a single sample and shaded according to the abundance of DGGE bands. The plot shows different bacterial community composition in the faeces from the patients and controls. The percentage of variation explained by each principal component is shown in brackets. (B) Band numbers and bacterial diversity obtained from the faecal profiles of CD patients. For each group, the mean values and standard deviation (bars) are shown. Bacterial diversity of CD patients was significantly lower compared with the controls. **, *p*<0.01.

To assess the changes of the composition and diversity in gut microbiota, the faecal samples from CD patients and healthy controls were subjected to DGGE analysis. The representative DGGE profiles were presented in Figure 1A. The dendrogram based on DGGE banding patterns showed low similarities ranging from 27% to 42% in faecal bacterial communities between CD patients and healthy controls. And CD group and the controls were separated into two different clusters (Fig. 1B). Principal component analyses, based on the abundance of DGGE bands, also confirmed that the bacterial community profiles were distinctly different for the patients and healthy individuals (Fig. 2A). The data demonstrated large differences in faecal microbiota composition of CD patients from the healthy controls.

The bacterial diversity, indicated as number of DGGE bands, was significantly reduced in CD patients compared with that in the controls (15.0±2.20 *vs.*19.9±2.58, *p* = 0.002) (Fig. 2B). The alternations of Shannon-Wiener diversity indices according to the number and intensity of bands were showed in Figure 2B. The mean diversity score was 2.19±0.13 for the patients and 2.58±0.21 for the healthy controls (*p* = 0.005), suggesting that the biodiversity of faecal microbiota in CD patients was decreased.

### The Predominant Bacterial Composition of the Faecal Microbiota in CD Patients

It has been known that the changes of intestinal bacterial composition likely contributed to the pathogenesis of CD in Western population [8]–[10]. Therefore, we further identified the alternations of the dominant bacterial species in the faeces of Chinese CD patients in search of the potential bacteria associated with CD of Chinese population. Eighty-four sequences of 16S rRNA gene fragments were obtained from both CD and healthy faecal samples. These genes fell into 25 different phylotypes based on the highest sequence similarity (97–100%) matched to GenBank sequences (Fig. 1A, Tables 1 and 2).

**Table 1:** The sequence analysis of DGGE bands from the faeces of the patients.

Band	Closest relative	Strain	GenBank accession	Identity (%)
F1	*Helicobacter canadensis*	MIT 98-5491 hc2	ACSF01000002	99
F2	*Acinetobacter lwoffii*	WJ10621	AFQY01000001	98
F3	*Enterococcus faecium*	TX0133a01	NZ_GL476170	98
F4	*Bacteroides sp.*	4_1_36	NZ_GL622512	98
F5	*Prevotella bryantii*	B14	NZ_ADWO01000048	98
F6	*Clostridium sp.*	M62/1	NZ_ACFX02000046	98
F7	*Faecalibacterium prausnitzii*	A2-165	NZ_ACOP02000011	100
F8	*Veillonella sp. oral taxon 158 str.*	F0412	NZ_AENU01000007	99
F9	*Salmonella enterica subsp. Enterica serovar Kentucky str.*	CVM29188	NZ_ABAK02000001	99
F10	*Ruminococcus flavefaciens*	FD-1	NZ_ACOK01000111	98
F11	*Clostridium bolteae*	ATCC BAA-613	NZ_ABCC02000039	97
F12	*Bacteroides pectinophilus*	ATCC 43243	NZ_ABVQ01000036	98
F13	*Clostridium leptum*	DSM 753	NZ_ABCB02000017	97
F14	*Shigella flexneri*	J1713	AFOW01000002	99
F15	*Escherichia coli*	PCN033	AFAT01000118	100
F16	*Bacteroides coprophilus*	DSM 18228	NZ_EQ973630	98
F17	*Veillonella parvula*	ATCC 17745	NZ_ADFU01000009	99
F18	*Enterobacteriaceae bacterium*	9_2_54FAA	NZ_ADCU01000033	97
F19	*Pseudoflavonifractor capillosus*	ATCC 29799	NZ_AAXG02000037	97
F20	*Veillonella atypica*	ACS-134-V-Col7a	NZ_AEDS01000059	100
F21	*Clostridium difficile*	6534	ADEJ01001157	100
F22	*Veillonella dispar*	ATCC 17748	NZ_ACIK02000004	99

**Table 2:** The sequence analysis of DGGE bands from the faeces of controls.

Band	Closest relative	Strain	GenBank accession	Identity (%)
H1	*Acinetobacter lwoffii*	WJ10621	AFQY01000001	98
H2	*Enterococcus faecium*	TX0133a01	NZ_GL476170	98
H3	*Bacteroides sp.*	4_1_36	NZ_GL622512	98
H4	*Prevotella bryantii*	B14	NZ_ADWO01000048	98
H5	*Roseburia intestinalis*	L1-82	NZ_GG692727	97
H6	*Clostridium sp.*	M62/1	NZ_ACFX02000046	98
H7	*Faecalibacterium prausnitzii*	A2-165	NZ_ACOP02000011	100
H8	*Bacillus coagulans*	36D1 ctg473	NZ_AAWV02000001	98
H9	*Veillonella sp. oral taxon 158 str.*	F0412	NZ_AENU01000007	99
H10	*Ruminococcus flavefaciens*	FD-1	NZ_ACOK01000111	98
H11	*Bacteroides pectinophilus*	ATCC 43243	NZ_ABVQ01000036	98
H12	*Bacteroides uniformis*	ATCC 8492	NZ_AAYH02000029	99
H13	*Bacteroides coprophilus*	DSM 18228	NZ_EQ973630	98
H14	*Pseudoflavonifractor capillosus*	ATCC 29799	NZ_AAXG02000037	98
H15	*Clostridium difficile*	6534	ADEJ01001157	100
H16	*Veillonella dispar*	ATCC 17748	NZ_ACIK02000004	99

**Figure 3:**
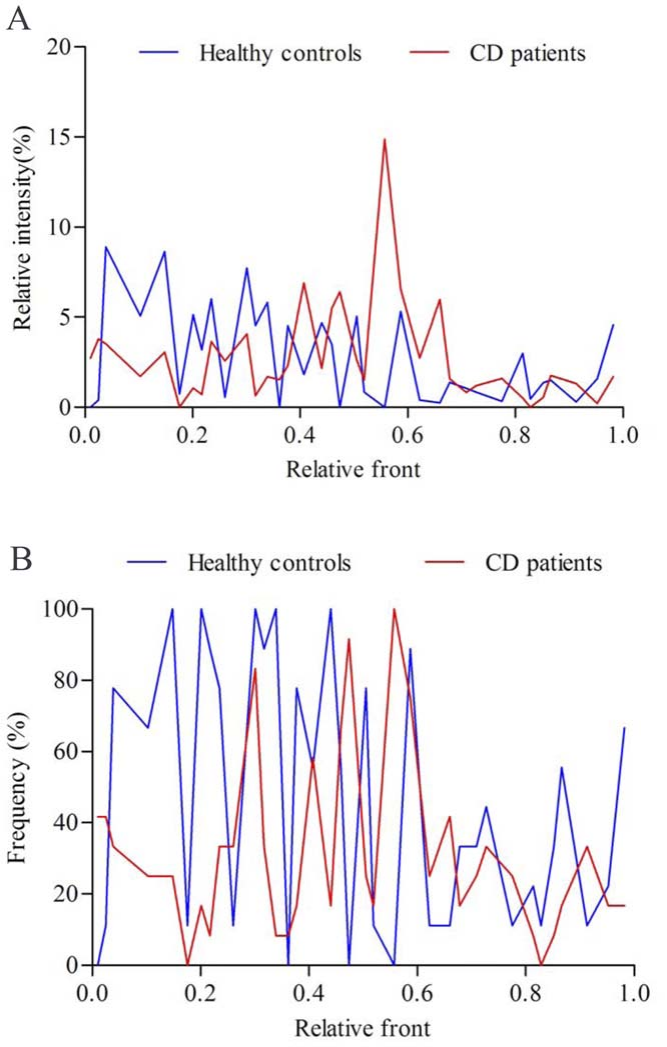
The relative abundance and frequency of predominant bacteria in faeces. (A) The mean richness of DGGE bands from the faecal samples. The staining intensity of fragments was expressed as a proportion (%) of the sum of all fragments in the same lane. (B) Frequency of DGGE fragments presented in CD group and the controls.

We quantified the relative abundance and frequency of fragments in DGGE profiles to identify which bacterial species might be involved in CD (Fig. 3). As shown in Fig. 3A and Table S1, the proportions of γ-Proteobacteria phylum, including *Escherichia coli* (F15), *Shigella flexneri* (F14), *Enterobacteriaceae bacterium* (F18) and *Salmonella enterica* (F9), were significantly increased in the patients as comparison with the control group (*p*<0.01). In contrast, most of Firmicutes bacteria detected in the faeces, such as *Faecalibacterium prausnitzii* (F7), *Clostridium sp.* (F6), *Ruminococcus flavefaciens* (F10), *Enterococcus faecium* (H2), *Roseburia intestinalis* (H5) and *Bacillus coagulans* (H8), showed a reduction in relative richness in the faecal microbiota of CD patients (*p*<0.01). However, *Clostridium bolteae* (F11) and *Clostridium leptum* (F13) were more abundant in CD patients than that in the controls (*p*<0.01). In addition, four bacteria of Bacteroidetes, *Bacteroides sp.* (H3), *Bacteroides pectinophilus* (H11), *Bacteroides uniformis* (H12) and *Prevotella bryantii* (H4) which dominated the faecal microbiota in the control group, were few present in CD patients (*p*<0.01). These results suggested that some opportunistic pathogenic γ-Proteobacteria greatly increased and became to be predominant in the faeces of CD patients instead of Bacteroides and Firmicutes. Analysis of sequence pools revealed significant differences in microbial community composition between CD patients and controls.

### The Mucosa-associated Microbiota in Ulcerated and Nonulcerated Areas

**Figure 4:**
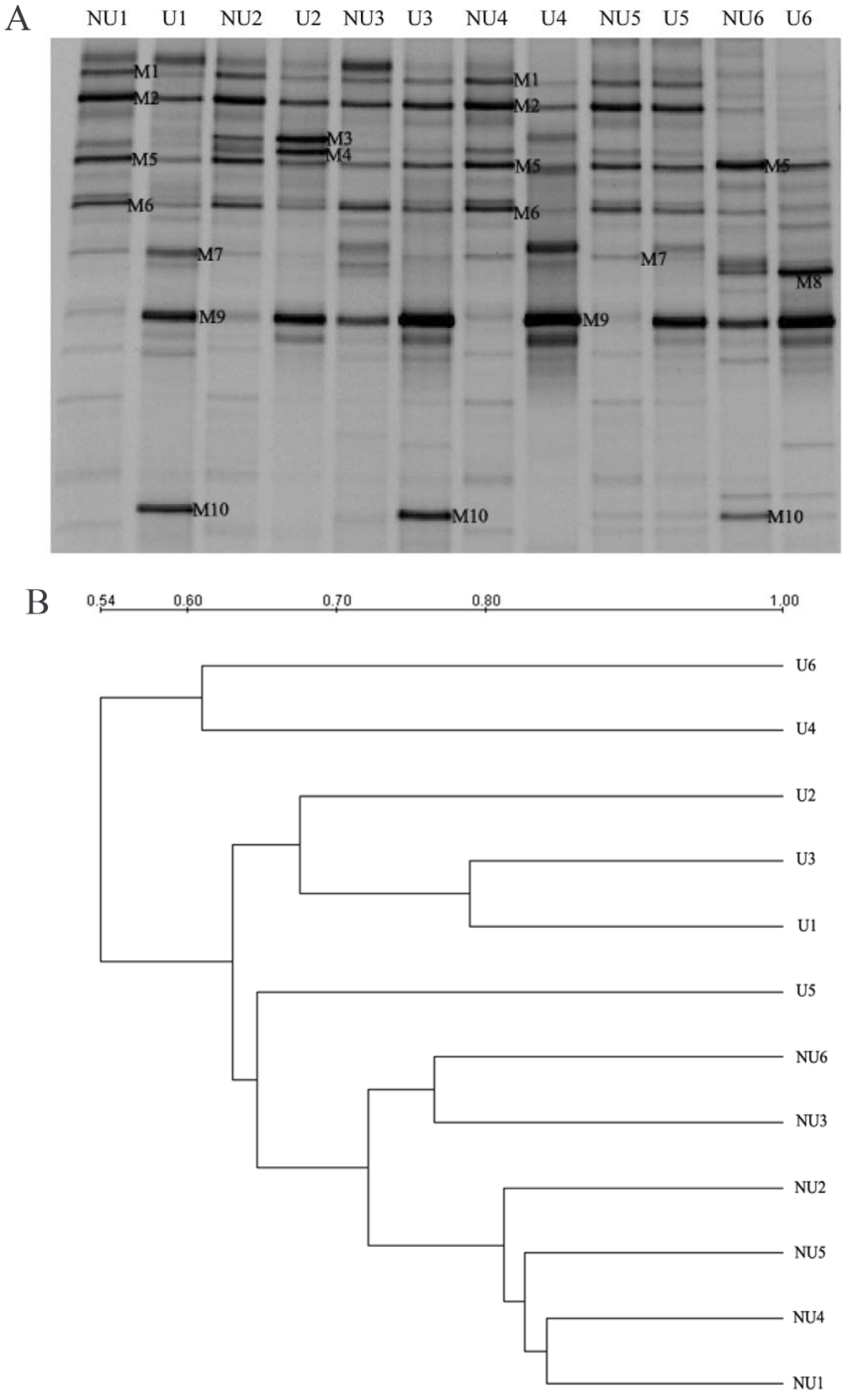
Comparison of the mucosa-associated microbiota between the ulcerated and nonulcerated ileum. (A) Molecular fingerprinting of the mucosal bacterial communities. U indicates the ulcerated ileal mucosa and NU is the nonulcerated ileal specimen. (B) Hierarchical distance clustering of mucosal DGGE profiles from CD patients. The similarities between mucosal specimens are shown in the dendrogram.

**Figure 5:**
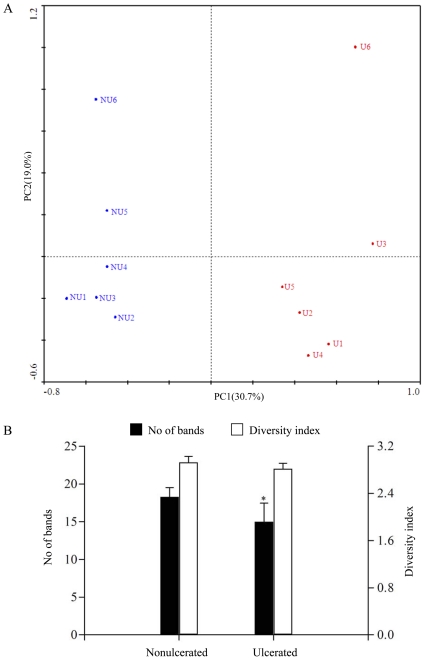
The plots of principal component analysis of microbial composition and diversity in mucosa-associated microbiota. (A) PCA plot of the mucosa-associated microbiota in CD patients. The plot demonstrated the difference of bacterial communities adhered in the ulcerated and nonulcerated sites. The percentage of variation explained by each principal component is shown in brackets. (B) Biodiversity of mucosal bacterial microbiota from the ulcerated and nonulcerated ileum. The mean values and standard deviation (bars) are shown. *, *p*<0.05.

It is important to note that the mucosal and faecal microbiota consist of distinct bacterial populations, and mucosa-associated microbes may be more important in CD pathogenesis [18], [19]. We profiled the bacterial composition and diversity in ileal mucosal specimens from the ulcerated and nonulcerated regions in CD patients (Fig. 4A). As shown in Figure 4B, the nonulcerated mucosal samples between different patients displayed higher similarities (>72%) and clustered together in the dendrogram. However, the banding patterns of the ulcerated mucosa were different from those in the nonulcerated tissues with lower similarities from 54% to 64%. The PCA plot clearly showed that the bacterial community composition was distinct between the ulcerated and nonulcerated specimens for both the first and the second principal component (Fig. 5A). Noticeably, the number of DGGE bands was fewer in the ulcerated mucosa (15.0±2.45) than that in the nonulcerated mucosa (18.3±1.21, *p* = 0.023) (Fig. 5B), suggesting a reduced diversity of bacterial community in the ulcerated areas in CD. Our results revealed that the mucosa-associated bacterial composition and diversity in the ulcerated regions was differentiated from the nonulcerated mucosa in CD.

### The Bacterial Phylotypes Adhered in Ulcerated and Nonulcerated Mucosa

**Figure 6:**
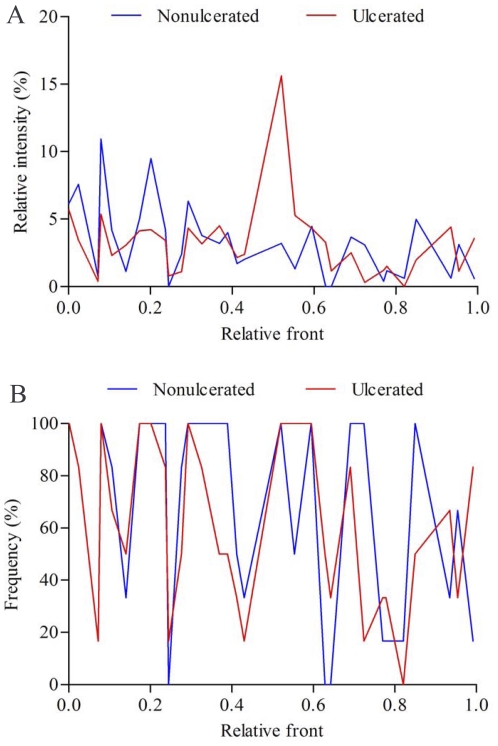
The relative richness and frequency of predominant microorganisms in mucosal specimens. (A) Changes in the relative abundance of fragments from the ulcerated and nonulcerated specimens. (B) The frequency of DGGE bands presented in the ulcerated and nonulcerated ileum.

**Figure 7:**
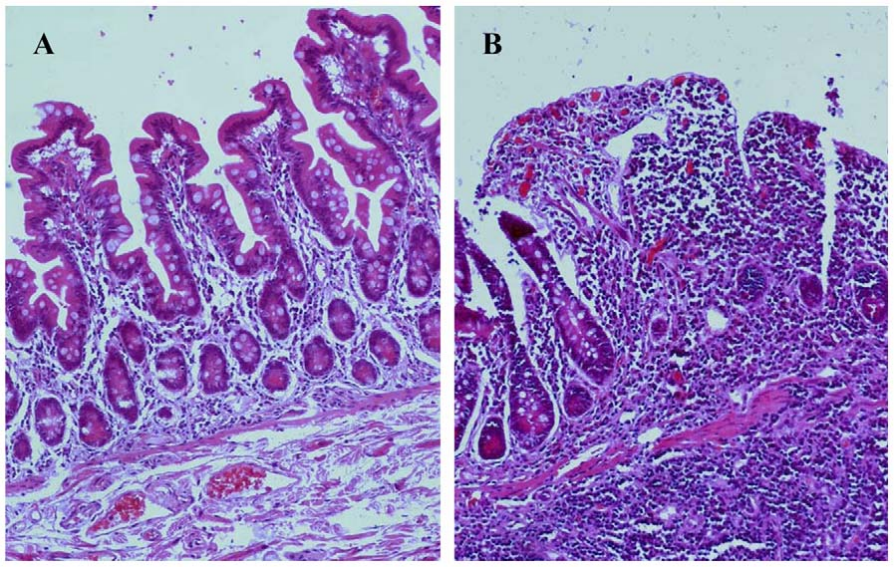
Histological characteristics of the ileal mucosa from CD patient. H&E staining showed normal epithelial architecture and few infiltrated inflammatory cells in the nonulcerated tissues (A). The mucosal specimen from ulcerated ileum revealed the disrupted mucosal architecture and the infiltration of inflammatory cells in (B). Magnifications: ×100.

For a better understanding of the potential involvement between the mucosa-associated microbiota and CD, we next sought to determine the shifts of predominant bacterial composition in the intestinal mucosa of CD individuals (Fig. 6). Ten bacterial phylotypes were identified using DNA sequencing and BLAST analysis (Fig. 4A, Table 3) and the significant alternations in the richness were found between the ulcerated and nonulcerated specimens (Fig. 6A, Table S2). We observed that the relative abundance of *Escherichia coli* (M9), *Clostridium difficile* (M10), *Clostridium sp.* (M3) and *Clostridium leptum* (M7) were much higher in the ulcerated group than that in the nonulcerated (*p*<0.01) (Fig. 6A, Table S2). The intensity of four bands corresponding to *Faecalibacterium prausnitzii* (M5), *Lactobacillus coleohominis* (M6), *Bacteroides sp.* (M2) and *Streptococcus gallolyticus* (M1), was decreased significantly in the ulcerated mucosa compared with that in the control regions (*p*<0.01). It indicated that the elevated proportion of some opportunistic pathogenic bacteria might be associated with the inflammatory response and the formation of mucosal ulceration in CD (Fig. 7).

**Table 3:** The sequence analysis of DGGE bands from mucosal samples.

Band	Closest relative	Strain	GenBank accession	Identity (%)
M1	*Streptococcus gallolyticus subsp. gallolyticus*	TX20005	NZ_AEEM01000001	98
M2	*Bacteroides sp.*	4_1_36	NZ_GL622512	98
M3	*Clostridium sp.*	M62/1	NZ_ACFX02000046	99
M4	*Bacillus coagulans*	36D1 ctg473	NZ_AAWV02000001	99
M5	*Faecalibacterium prausnitzii*	A2-165	NZ_ACOP02000011	100
M6	*Lactobacillus coleohominis*	101-4-CHN	NZ_GG698808	98
M7	*Clostridium leptum*	DSM 753	NZ_ABCB02000017	98
M8	*Yersinia enterocolitica subsp. palearctica*	YO527	CACW01000009	98
M9	*Escherichia coli*	PCN033	AFAT01000118	100
M10	*Clostridium difficile*	6534	ADEJ01001157	100

### Phylogenetic Analysis of the Predominant Bacteria

**Figure 8:**
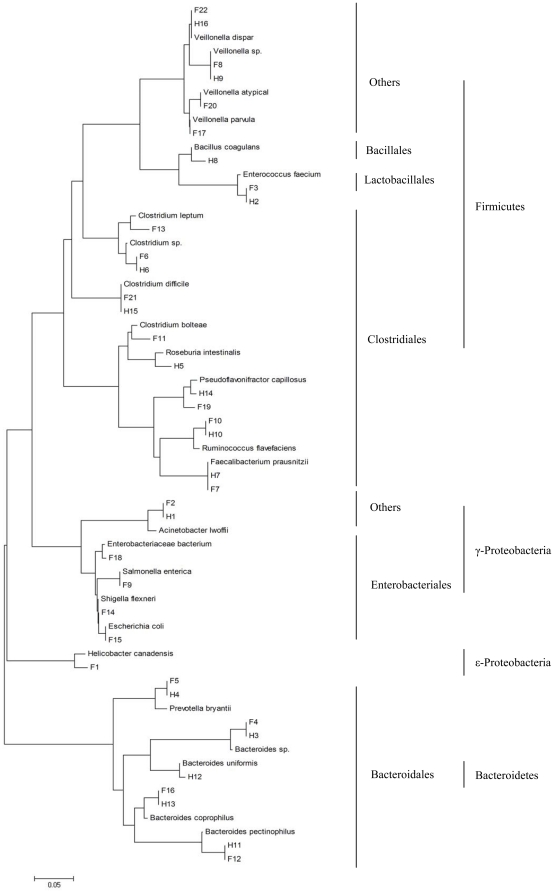
Phylogenetic tree generated from the 16S rDNA sequences of predominant bacterial species in the faeces. The sequences were aligned with closely related 16S rDNA sequences retrieved from GenBank database using the BLAST. The dominant sequences from the faeces of CD patients and healthy subjects were divided into 4 clusters: Firmicutes, Bacteroidetes, γ-Proteobacteria and ε-Proteobacteria. The scale bar represents the genetic distance.

The sequences representing the respectively excised DGGE bands were phylogenetically analyzed by ClustalW and neighbor-joining alignment. The phylogenetic dendrogram showed that the dominant sequences from the faeces in CD patients were divided into 4 clusters (Fig. 8): Firmicutes (total 12 sequences: 7 of Clostridiales; 1 of Lactobacillales, 4 of others), Bacteroidetes (total 4 sequences, 4 of Bacteroidales), γ-Proteobacteria (total 5 sequences: 4 of Enterobacteriales, 1 of others) and ε-Proteobacteria (1 sequence). In the healthy controls, the sequences were belonged to 3 clusters: Firmicutes (total 10 sequences: 6 of Clostridiales; 1 of Lactobacillales, 1 of Bacillales, 1 of others), Bacteroidetes (total 5 sequences: 5 of Bacteroidales), γ-Proteobacteria (1 sequence, 1 of Enterobacteriales). It revealed that the predominant bacterial composition in the faeces was altered and the Proteobacteria phylum was overrepresented in patients with CD.

**Figure 9:**
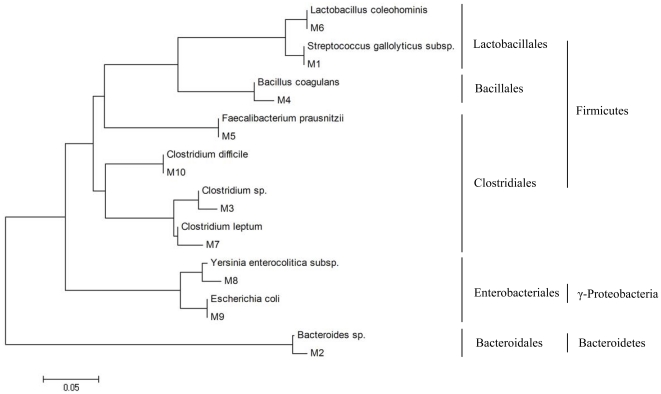
Phylogenetic relationship of 16S rDNA sequences obtained from the mucosal specimens of CD patients. The sequences were aligned with reference strains obtained from the GenBank database. Ten predominant bacterial species from the mucosal specimens were phylogenetically clustered into 3 phylum: Firmicutes, Bacteroidetes and γ-Proteobacteria. The scale bar represents the genetic distance.

Ten dominant sequences obtained from the mucosal specimens were phylogenetically clustered into 3 phylum (Fig. 9): Firmicutes (total 7 sequences: 4 of Clostridiales; 2 of Lactobacillales, 1 of Bacillales), Bacteroidetes (Bacteroidales: 1 sequence) and γ-Proteobacteria (2 sequences, 2 of Enterobacteriales:). The dominant mucosa-associated microbiota showed greater differences from the dominant fecal microbiota in CD

patients.

## Discussion

In this study, we used culture-independent techniques to characterize the intestinal microbiota dysbiosis in the faeces and ileal mucosa of Chinese patients with CD. Cluster analysis of the bacterial profiles in faecal samples showed distinct clustering of CD patients and controls. The biodiversity of faecal microbiota was reduced in CD patients. The dysbiosis signature we found in Chinese patients with CD was markedly characteristic by an increase of the richness of some opportunistic pathogenic γ-Proteobacteria (especially *Escherichia coli* and *Shigella flexneri*) and a decrease of the proportion of Firmicutes and Bacteroides. The profiles of the mucosa-associated microbiota in CD patients demonstrated that the predominant composition of bacteria attached to the ulcerated mucosa was distinguishable from that in the nonulcerated area. The diversity of the microbiota in the ulcerated sites was decreased in comparison to that in the nonulcerated mucosa. The relative abundance of *Escherichia coli* and *Clostridium* spp. were found to be much higher in the ulcerated mucosa than that in the nonulcerated. *Faecalibacterium prausnitzii*, *Lactobacillus coleohominis*, *Bacteroides sp.* and *Streptococcus gallolyticus* were decreased significantly in the richness in the ulcerated mucosa. Our study demonstrated the molecular characteristic of the intestinal microbiota dysbiosis in the faeces and the mucosal tissues in Chinese population with CD.

Previous studies have provided evidence of the roles of the intestinal microbiota in the development and/or progression of CD [8]–[10]. The intestinal microbiota of patients with both active and inactive CD differs from that of healthy subjects [20]. However, the dysbiosis of intestinal bacterial community in Chinese CD patients is still unknown. In the present study, we profiled the bacterial composition and diversity of the fecal samples obtained from Chinese patients with CD. Large differences in the fecal microbiota were presented between the individuals with CD and healthy controls. We showed a reduced bacterial diversity in CD individuals compared to healthy controls, which was consistent with numerous other reports in Western populations with CD [14], [15]. This is the first finding that specifically pointed to a lower diversity for Chinese population subjects with CD.

Sequence analysis of 16S rRNA gene fragments provided the detailed description of the bacterial composition in the faeces of CD patients. Our results showed that the dominant bacterial species were altered in the faeces of CD patients, which was characterized by an overrepresentation of γ-Proteobacteria and a relative lack of Firmicutes and Bacteroidetes phylum. We observed that *Escherichia coli*, a major member of γ-Proteobacteria, had a significantly elevated abundance and became to be dominant in the faeces from CD patients. This finding corroborated recent researches suggesting a possible link between *Escherichia coli* and the pathogenesis of CD [21]–[23], [40], [41]. Our another important finding was a decreased proportion of Firmicutes phylum, in particular of *Faecalibacterium prausnitzii* in the faecal microbiota of CD patients. *Faecalibacterium prausnitzii* has been known as a potent anti-inflammatory commensal organism [42]. Our data suggested that *Faecalibacterium prausnitzii* might be negatively associated with CD, which was consistent with the results of previous studies [24], [25], [42]. The other members of Firmicutes, including *Clostridium sp. Ruminococcus flavefaciens*, *Enterococcus faecium*, *Roseburia intestinalis* and *Bacillus coagulans* were also observed markedly decreased in the faeces in CD patients. These Gram positive anaerobic bacteria usually account for a major fraction of the faecal microbiota in healthy subjects [31]. The data indicated that a reduced richness of Firmicutes might be implicated in the pathogenesis of CD. In addition, the quantitative composition of *Bacteroides* spp. population was significantly decreased in CD patients compared with the normal controls. Seksik *et al.* also found a decreased population level of Bacteroidetes in the faeces from CD patients [17]. A group-specific molecular analysis showed a significant low diversity of Bacteroides in the faecal microbiota of CD patients [18], [19]. However, the studies of Bacteroidets in CD are still controversial. Several culture studies have shown a possible increase of *Bacteroides* spp. population level in CD [43], [44].

Apart from that, we demonstrated some unique characteristics of faecal bacterial imbalance in Chinese CD patients. We found that *Shigella flexneri* was more prevalent and abundant in CD patients but not present in healthy individuals. Some opportunistic pathogenic Proteobacteria such as *Enterobacteriaceae bacterium*, *Salmonella enterica* and *Helicobacter Canadensis* were also found occasionally in CD patients. These bacterial species were all absent from the previous investigation of CD. In addition, an elevated richness of *Clostridium bolteae* and *Clostridium leptum* was detected in CD patients and it was not consistent with the previous results. It was usually thought that the Clostridium phylogenetic group, especially *Clostridium leptum* subgroup, was low abundant in CD patients compared with the healthy subjects [13], [17]. *Veillonella dispar* and *Veillonella sp.* that was abundant in healthy controls were absent from the dominant biota of CD patients (*p*<0.01; Fig. 1A, Table S1), which was few involved in CD in previous studies. These findings appeared different from the results obtained from Western subjects with CD. It has been known that the composition of intestinal microbiota is influenced by genetic background and food custom [31], [32], [45], [46]. Previous study has reported that the variants of NOD2 and other IBD genes in Chinese CD patients were distinct from Western CD patients [47]. So it was probable that human genetic background might be responsible for the differences of intestinal microbiota in Chinese patients. Furthermore, different dietary habits might be another important reason for the variations of intestinal microbiota between Chinese and Western subjects [48], [49]. Our current data might provide new bacterial species linked to active CD in China.

A relationship between disturbed microbiota and mucosal inflammation has not been established. Because mucosa-associated microbial species is close proximity to the host epithelium, the bacteria attached in intestinal mucosa are more likely to be involved in the inflammatory response of the gut than the luminal counterparts [18], [19]. It has been recognized that the intestinal mucosa-associated microbiota differs substantially from the fecal microbiota [16], [18]. However, the mucosa-associated microbiota in CD is poorly known, especially for inflammatory settings in CD patients. In this study, we used molecular phylogenetic analysis to characterize the mucosal microbiota and compared the differences of predominant bacterial composition between the ulcerated and nonulcerated tissues in the patients with ileal CD. As expected from previous studies of the mucosa-associated microbiota in Western CD subjects [18]–[20], we observed a decreased biodiversity of bacteria biota in the ulcerated mucosa in Chinese patients. The dominant bacterial structures in the ulcerated and the nonulcerated tissues showed low similarity, which were not in agreement with previous work [50]. Although the prevalence of the predominant microbial species were similar (Fig. 6B), their relative abundance was shifted in the ulcerated mucosa as compared with the nonulcerated areas (Fig. 6A), which was a major contributor for the lower similarity between the two different sites. The quantitative analysis of 16S rDNA fragments showed that *Escherichia coli* was more abundant in the ulcerated sites of intestinal mucosa than that in the nonulcerated mucosa. Increased abundance of *Escherichia coli* in biopsies from CD patients has previously been reported [13], [16], [19], [21]–[24]. These findings indicated that *Escherichia coli* was specifically associated with ulceration in CD patients [21]–[23]. Similar to previous results obtained from Western CD patients [24], [25], *Faecalibacterium prausnitzii* were less dominant in ulcerated mucosa in CD patients. In addition, the relative richness of *Bacteroides sp.*, *Lactobacillus coleohominis* and *Streptococcus gallolyticus* were decreased significantly in the ulcerated mucosa, which was few detected in mucosa-associated microbiota in the previous study. The decreased richness of these bacterial species may be resulted from the overgrowth of the pathogens attached in the ulcerated sites. These differences between the ulcerated and the nonulcerated mucosa may reflect the altered nature of the mucus present on the mucosal surface of CD patients. The bacterial dysbiosis in the ulcerated mucosa of CD patients may be closely associated to the inflammatory response and disrupted mucosal structure seen in CD patients (Fig. 7).

In summary, we reported the results of a prospective study on intestinal microbiota in Chinese patients with CD. The present study defined for the first time the molecular characteristics of intestinal microbiota dysbiosis in Chinese CD subjects. We identified a reduced biodiversity of faecal bacterial community in CD. The faecal bacterial dysbiosis in CD patients was characterized by an increase of the richness γ-Proteobacteria (especially *Escherichia coli* and *Shigella flexneri*) and a reduced proportion of Bacteroides and Firmicutes. For mucosa-associated microbiota, we demonstrated a decrease of bacterial diversity in the ulcerated mucosa compared with that in the nonulcerated sites. In addition, the quantitative composition of the bacteria adhered to the ulcerated areas was different from that in the nonulcerated mucosa, which was mainly characteristic by an elevated *Escherichia coli* level and a reduction of *Faecalibacterium prausnitzii*, *Lactobacillus coleohominis*, *Bacteroides sp* and *Streptococcus gallolyticus* in the ulcerated sites. Taken together, we demonstrated an overrepresentation of some opportunistic pathogenic bacteria and a decrease in beneficial bacterial species in intestinal microbiota of Chinese CD patients, which indicated a possible relationship between some bacteria and the origin of CD. These findings may provide new clues into determining the aetiological mechanisms of CD in China.

## Supporting Information

Table S1 **Differences of the predominant bacterial species in the faeces from CD patients and healthy controls.**
(DOC)

Table S2 **Differences of the predominant bacterial species adhered in the ulcerated and nonuclerated mucosa.**
(DOC)
