# Supplementary material for: Molecular-Phylogenetic Characterization of the Microbiota in Ulcerated and Non-Ulcerated Regions in the Patients with Crohn's Disease
Source: PLoS One. 2012 Apr 18;7(4):e34939. doi: 10.1371/journal.pone.0034939 (PMC3329531; doi:10.1371/journal.pone.0034939)
Supplement: Table S1 — Differences of the predominant bacterial species in the faeces from CD patients and healthy controls. (DOC) [file pone.0034939.s001.doc]

**Supplementary Table 1 Differences of the predominant bacterial species in the faeces from CD patients and healthy controls**

| Closest species | Relative  front | Frequency (%) | | Relative richness (%) | | Phylum |
| --- | --- | --- | --- | --- | --- | --- |
|  |  | HC (n=9) | CD (n=12) | HC (n=9) | CD (n=12) |  |
| *Escherichia coli* | 0.557 | 0.0 | 100.0 | 0.00 | 14.87** | γ-Proteobacteria |
| *Shigella flexneri* | 0.474 | 0.0 | 91.7 | 0.00 | 6.39** |  |
| *Enterobacteriaceae bacterium* | 0.660 | 11.1 | 41.7 | 0.25 | 5.98** |  |
| *Salmonella enterica subsp. enterica serovar Kentucky str.* | 0.362 | 0.0 | 8.3 | 0.00 | 1.53** |  |
| *Acinetobacter lwoffii* | 0.039 | 77.8 | 33.3 | 8.90 | 3.52** |  |
| *Helicobacter canadensis* | 0.010 | 0.0 | 41.7 | 0.00 | 2.72** | ε-Proteobacteria |
| *Clostridium bolteae* | 0.407 | 55.6 | 58.3 | 1.82 | 6.89** | Firmicutes |
| *Clostridium leptum* | 0.459 | 55.6 | 66.7 | 3.50 | 5.49** |  |
| *Faecalibacterium prausnitzii* | 0.301 | 100.0 | 83.3 | 7.72 | 4.07** |  |
| *Clostridium sp.* | 0.235 | 77.8 | 33.3 | 6.00 | 3.65** |  |
| *Roseburia intestinalis* | 0.217 | 88.9 | 8.3 | 3.20 | 0.71** |  |
| *Clostridium difficile* | 0.866 | 55.6 | 16.7 | 1.49 | 1.75 |  |
| *Ruminococcus flavefaciens* | 0.377 | 77.8 | 16.7 | 4.53 | 2.31** |  |
| *Pseudoflavonifractor capillosus* | 0.727 | 44.4 | 33.3 | 0.84 | 1.20 |  |
| *Bacillus coagulans* | 0.317 | 88.9 | 33.3 | 4.55 | 0.64** |  |
| *Enterococcus faecium* | 0.103 | 66.7 | 25.0 | 5.05 | 1.70** |  |
| *Veillonella dispar* | 0.982 | 66.7 | 16.7 | 4.59 | 1.69** |  |
| *Veillonella parvula* | 0.622 | 11.1 | 25.0 | 0.40 | 2.74** |  |
| *Veillonella atypica* | 0.775 | 11.1 | 25.0 | 0.34 | 1.60** |  |
| *Veillonella sp. oral taxon 158 str.* | 0.339 | 100.0 | 8.3 | 5.81 | 1.69** |  |
| *Bacteroides sp.* | 0.148 | 100.0 | 25.0 | 8.63 | 3.06** | Bacteroidetes |
| *Prevotella bryantii* | 0.201 | 100.0 | 16.7 | 5.12 | 1.07** |  |
| *Bacteroides pectinophilus* | 0.440 | 100.0 | 16.7 | 4.70 | 2.17** |  |
| *Bacteroides uniformis* | 0.505 | 77.8 | 25.0 | 5.02 | 2.62** |  |
| *Bacteroides coprophilus* | 0.587 | 88.9 | 75.0 | 5.31 | 6.54* |  |

HC: Healthy controls; CD: Crohn’s disease patients; * *p*<0.05; ** *p*<0.01
