# Supplementary material for: Molecular-Phylogenetic Characterization of the Microbiota in Ulcerated and Non-Ulcerated Regions in the Patients with Crohn's Disease
Source: PLoS One. 2012 Apr 18;7(4):e34939. doi: 10.1371/journal.pone.0034939 (PMC3329531; doi:10.1371/journal.pone.0034939)
Supplement: Table S2 — Differences of the predominant bacterial species adhered in the ulcerated and nonuclerated mucosa. (DOC) [file pone.0034939.s002.doc]

**Supplementary Table 2 Differences of the predominant bacterial species adhered in the ulcerated and nonuclerated mucosa**

| Closest species | Relative  front | Frequency (%) | | Relative richness (%) | | Phylum |
| --- | --- | --- | --- | --- | --- | --- |
|  |  | NU (n=6) | U (n=6) | NU (n=6) | U (n=6) |  |
| *Escherichia coli* | 0.520 | 100.0 | 100.0 | 3.19 | 15.61** | γ-Proteobacteria |
| *Yersinia enterocolitica subsp. palearctica* | 0.430 | 33.3 | 16.7 | 2.02 | 2.38 |  |
| *Clostridium difficile* | 0.935 | 33.3 | 66.7 | 0.62 | 4.42** | Firmicutes |
| *Clostridium sp.* | 0.140 | 33.3 | 50.0 | 1.12 | 3.06** |  |
| *Clostridium leptum* | 0.369 | 100.0 | 50.0 | 3.19 | 4.49* |  |
| *Faecalibacterium prausnitzii* | 0.201 | 100.0 | 100.0 | 9.48 | 4.20** |  |
| *Streptococcus gallolyticus subsp. gallolyticus* | 0.024 | 83.3 | 83.3 | 7.57 | 3.44** |  |
| *Lactobacillus coleohominis* | 0.293 | 100.0 | 100.0 | 6.33 | 4.33** |  |
| *Bacillus coagulans* | 0.174 | 100.0 | 100.0 | 5.02 | 4.12* |  |
| *Bacteroides sp.* | 0.079 | 100.0 | 100.0 | 10.93 | 5.36** | Bacteroidetes |

NU: nonulcerated mucosa; U: ulcerated mucosa; * *p*<0.05; ** *p*<0.01
